# Advantages and disadvantages of using Carbon Nanostructures in Reproductive Medicine: two sides of the same coin

**DOI:** 10.5935/1518-0557.20210070

**Published:** 2022

**Authors:** Hadi Zare-Zardini, Nooshin Hatamizadeh, Navid Haddadzadegan, Hossein Soltaninejad, Mojgan Karimi-Zarchi

**Affiliations:** 1 Hematology and Oncology Research Center, Shahid Sadoughi University of Medical Sciences, Yazd, Iran; 2 Medical Nanotechnology and Tissue Engineering Research Center, Yazd Reproductive Sciences Institute, Shahid Sadoughi University of Medical Sciences, Yazd, Iran; 3 Department of Biomedical Engineering, Meybod University, Meybod, Iran; 4 Department of Obstetrics and Gynecology, Shahid Sadoughi University of Medical Sciences, Yazd, Iran; 5 Department of Anesthesiology and Critical Care, Shahid Sadoughi University of Medical Sciences, Yazd, Iran; 6 Department of Nanobiotechnology, Faculty of Biological Sciences, Tarbiat Modares University, Tehran, Iran; 7 Department of Gynecology Oncology, Firoozgar Hospital, Iran University of Medical Sciences, Tehran, Iran

**Keywords:** carbon nanostructures, reproductive medicine, side effects, attention

## Abstract

Carbon nanostructures are important nanomaterial with interesting physical and chemical properties. These nanostructures have been assessed for application in different fields of medicine, such as cancer detection and treatment, Parkinson disease, reproductive medicine, etc. This nanomaterial can be used in reproductive medicine as a drug delivery system, antifungal, antiviral, and antibacterial agent, condom-coating agent, enhancer of sperm fertilizing ability, ectopic pregnancy treatment, trophoblastic diseases, endometriosis, uterine fibroids, and Assisted Reproduction Techniques (ART) improvement. The other side of this coin involves various side effects of carbon nanostructures, especially negative effects on reproductive systems. All carbon nanostructures showed toxicity on the reproductive system by producing reactive oxygen species and oxidative stress. Less attention has been given to the unique properties of carbon nanostructures, except for their practical attractiveness, the other side of this coin, namely the risks and side effects of these compounds - especially in the case of a reproductive system that supports the survival and health of future generations. Therefore, we suggest paying particular attention to the negative aspects of the increasing use of carbon nanostructures.

## BACKGROUND

Application of different nanostructures (nanocages, magnetic nanochains, nanocomposites, nanofabrics, nanofibers, nanoflowers, nanofoams, nanoholes, nanomesh, nanopillars, nanopin film, nanoplatelet, nanoribbon, nanoring, nanorod, nanosheet, nanoshell, nanotip, nanowires, nanostructured film, quantum dot, lipid nanostructures, and etc.) has been developed in different fields over the past years, especially in medicine (Zahid *et al*., 2013; Zare-Zardini *et al*., 2013; 2015; 2016; 2018a; 2018b; 2020). Carbon nanostructures with various shapes (graphene and its derivatives, SWCNTs, MWCNTS, and fullerene) have amazing physical and chemical properties including thermal and electrical conductivity, vibroelectronic properties, high aspect ratio, strength and elasticity, electron emission, high tensile strength, high flexibility, and etc. so, due to these properties, carbon nanostructures can be used in the field of medicine (Jeevanandam *et al*., 2018; Mukhin *et al*., 2015; Patel *et al*., 2020).

One side of the coin, e.g. the use of carbon nanostructures in reproductive medicine, has been the subject of many studies. Effective and positive mechanisms of these nanostructures in various fields of reproductive medicine, such as increasing sperm fertility, prevention of unplanned pregnancy, increasing sperm and egg retention in culture medium, preventing the transmission of sexually transmitted infections, increasing the effectiveness of genital health drugs such as chemotherapy in the treatment of reproductive system cancers, and etc., has been well studied and proven, and the most detailed topics related to these positive effects have been discussed in many papers The use of carbon nanostructures in ART include delivery system for increase bioavailability and permeability of sexual hormones, gametes and embryos selection, and gene transfer. In the field of embryogenesis, these nanostructures can be used for labelling pre-implanted embryos, Nano nutrition, and gene therapy. Carbon nanostructures have been used in reproductive oncology for treatment and diagnostic purposes. Infection detection, targeted treatment, microbicides and vaccine design for prevention, are all applications of Carbon nanostructures in the field of reproductive infections. These nanostructures can be used as alternative for surgical intervention in ectopic pregnancy, trophoblastic diseases, endometriosis and uterine fibroids (Azizi-Lalabadi *et al*., 2020; Bernabò *et al*., 2019; 2020; George *et al*., 2018; He *et al*., 2013; Kang *et al*., 2007; Krishnaraj *et al*., 2014; Liu *et al.,* 2017; Liu & Chen, 2016; Maleki Dizaj *et al*., 2015; Ramal-Sanchez *et al*., 2019; Saliev, 2019; Zare-Zardini *et al*., 2020).

**Figure 1:**
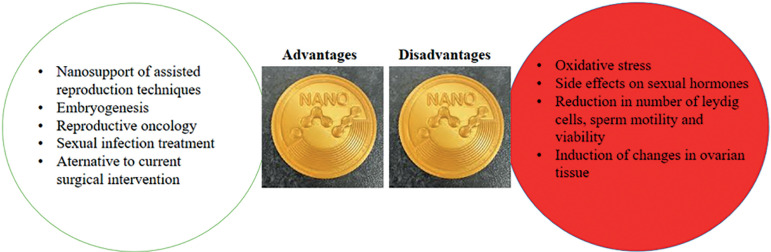
The two sides of the coin for using carbon nanostructures in reproductive medicine.

However, attention to the other side of this coin, namely the risks for increasing the use and the range of carbon nanostructures, especially their toxicity on the reproductive system, has been neglected. Compared to the positive aspects of the use of carbon nanostructures in reproductive medicine, much less attention has been paid to its negative aspects (Zare-Zardini *et al*., 2018a; 2018b) (Figure 1). A comparison of the number of published papers in both fields and the significant differences in these areas prove this claim. Carbon nanostructures have different side effects on normal cells, tissues and organs. All shapes of these nanostructures showed adverse toxicity on reproductive systems. Among carbon nanostructures, it has been proven that the highest side effects belong to SWCNTs. The lowest toxicity was seen in the application of Fullerene. Functionalization of graphene with active functional groups such as -COOH and -NH2 lead to increased toxicity on the reproductive system. Reduced graphene had the lowest toxicity among all types of graphene. There are limited studies on the mode of negative impacts of these nanostructures on the reproductive system, but these limited studies have shown that the main acceptable mechanism in the toxicity of carbon nanostructures on the reproductive system is the production of reactive oxygen species and oxidative stress, side effects on sexual hormones, reduction in the number of Leydig cells, sperm motility and viability, and induction of changes in ovarian tissue, such as change in the expression of genes involved in ovarian hormones and cytokine pathways, disruption of balanced hormone levels by effect on hypothalamic-pituitary-gonadal axis or stimulation of secretory cells, reduction of steroidogenesis in the ovary, and so on. The negative effects of carbon nanostructures on the male reproductive system are greater than on the female reproductive system due to lack of suitable protection mechanisms for the testes (Asghar *et al*., 2016; Dehghanbaghi & Zare-Zardini, 2019; Xu *et al*., 2016; Vasyukova *et al*., 2015).

Due to less attention to the negative aspects regarding the use of carbon nanostructures, we suggest that in future studies and research, more attention be paid to other aspects, namely the harmful effects related to carbon nanostructures. Due to the importance of reproductive system health for survivors and the health of born children, it is highly important to pay attention to the negative impact of carbon nanostructures on the reproductive system.
